# Bio-inspired strategies for designing antifouling biomaterials

**DOI:** 10.1186/s40824-016-0064-4

**Published:** 2016-06-20

**Authors:** Vinod B. Damodaran, N. Sanjeeva Murthy

**Affiliations:** New Jersey Center for Biomaterials, Rutgers – The State University of New Jersey, Piscataway, NJ 08854 USA

**Keywords:** Antifouling, Bio-inspired, Biomaterials, Nitric oxide, Hydration, Peptoids, PEG, DOPA, Zwitterions, Micropatterning

## Abstract

Contamination of biomedical devices in a biological medium, biofouling, is a major cause of infection and is entirely avoidable. This mini-review will coherently present the broad range of antifouling strategies, germicidal, preventive and cleaning using one or more of biological, chemical and physical techniques. These techniques will be discussed from the point of view of their ability to inhibit protein adsorption, usually the first step that eventually leads to fouling. Many of these approaches draw their inspiration from nature, such as emulating the nitric oxide production in endothelium, use of peptoids that mimic protein repellant peptides, zwitterionic functionalities found in membrane structures, and catechol functionalities used by mussel to immobilize poly(ethylene glycol) (PEG). More intriguing are the physical modifications, creation of micropatterns on the surface to control the hydration layer, making them either superhydrophobic or superhydrophilic. This has led to technologies that emulate the texture of shark skin, and the superhyprophobicity of self-cleaning textures found in lotus leaves. The mechanism of antifouling in each of these methods is described, and implementation of these ideas is illustrated with examples in a way that could be adapted to prevent infection in medical devices.

## Background

Biofouling is the contamination of surfaces by microbes that include bacteria (prokaryotes), fungi and viruses. In medical applications, biofouling occurs on surgical equipment, protective apparel, packaging, guide wires, sensors, prosthetic devices, and medical implants, and most familiarly on catheters, drug delivery devices and contact lenses. Microbial contamination, and the subsequent risk of infection, biosensor failure and implant rejection, is a major driver in developing efficient antifouling strategies [1]. According to a recent study initiated by the Centers for Disease Control and Prevention, about 26 % of the health-care related infections are caused by these device-associated infections in U.S. acute care hospitals alone in 2011 [2]. Prevention of morbidity and mortality associated with biofilm-mediated infections calls for the replacement of contaminated devices, as well as treatment with antibiotics, which sometimes may be ineffective [3].

Since biofouling is mediated by proteins, inhibition of protein adsorption prevents the cause of infection at the source. The layer of adsorbed protein on surfaces serves as a platform for cell attachment, and subsequent bacterial colonization that leads to the formation of bacterial films [4]. Protein fouling is a major challenge in the development of numerous blood-contacting biomedical devices caused by the nonspecific adhesion of biological components including proteins to the device surface. These pro-inflammatory processes result in thrombus formation, which leads to platelet formation, and ultimately to device failures and fatal complications. There are many excellent reviews [5–7] on antifouling strategies for a wide variety of applications, marine, industrial (e.g., reverse osmosis membranes), and biomedical applications [8–12]. We distinguish our review from these by focusing on biomedical devices and discuss a broad range of possible methods, biological, chemical and physical, in sufficient detail.

## Strategies for making antifouling biomedical surfaces

There are three strategies: First is the use of biocides, antibacterials that kill bacteria or antimicrobial that kill bacteria and other microorganisms. Second is to repel the proteins, and subsequently cells, and thus prevent biofouling. Third is to create surfaces that self-clean so that the organisms do not remain attached. Nature appears to have developed a combination of these approaches.

The methods that are currently used or in conceptual stages create antifouling surfaces on blood-contacting biomedical devices either chemically modify the surface composition or physically alter the surface topography. These methods alter the hydration layer at the surface and thereby inhibit the adsorption of proteins. Exceptions are the nitric oxide (NO)-based technique that kills the cells and peptide/peptoid-based methods that attempt to prevent cells for adhering to the surface. In this review, we will discuss the key strategies for inhibiting protein adsorption on biomedical devices and emphasize the principle, mechanisms and illustrate them with a few applications. In the following sections we will review two chemical techniques, one based on impregnating the surface with biological molecules and the other that incorporates biologically active synthetic components onto the device’s surface, and two physical techniques in which antifouling is achieved by micropatterning. These are listed in Table 1.

**Table 1:** Comparison of various antifouling strategies presented in this paper

Antifouling strategy	Principle/Mechanism	Advantages	Disadvantages
1. Biological molecules			
1.1 Nitric oxide-releasing materials	Oxidative or nitrosative stress-inducing moieties are produced within the biofilm structure to cause bacteriophage induction, and cell lysis.	Synthetic NO donor supplements the natural sources	Because of high reactivity (instability), storage and delivery requires special attention.
			Selective to only certain bacterial types.
1.2 Peptide and peptoid modified surfaces	Through structural reformations that inhibit cell adhesion.	Exceptional resistance to a wide variety of proteins.	High cost of peptide/peptoid modification of surfaces
		Tailorable surface structure for optimum performance.	
2. Chemical modification			
2.1 Hydrophilic polymers	A layer of strongly bound water that cannot be displaced by a protein and thus inhibiting protein adsorption.	Uses poly(ethylene glycol) (PEG), an U.S. federal drug administration (FDA) approved GRAS (generally recognized as safe) substance	Oxidative damages and low surface densities limit long-term application.
2.2 Immobilization of PEG	Anchoring of PEG using a mussel-mimicking linker.	Less susceptible to hydrolytic degradation than free PEG	Limited by the availability of suitable surface functionalities for anchoring PEG
2.3 Zwitterionic polymers	High protein resistance through the formation of “super-hydrophilicity”.	Long-term antifouling characteristics.	Limited commercial availability of zwitterionic polymers
		Unique capability for ligand immobilization.	
2.4 Hydrophobic polymers	Inhibits the adsorption of proteins that require polar surfaces.	Many hydrophobic polymers are commercially available.	Toxicity concerns with many hydrophobic polymers.
3. Micropatterning of surfaces			
3.1 Lotus-effect	Self-cleaning ability of the superhydrophobic surface prevents adhesion	A physical texture enhances the role of the simple waxy surface	Limited by fabrication techniques and general applicability
3.2 Shark-skin patterns	Surface patterns along with an antifouling chemical agent sloughs of attached cells	A physical modification of the surface to enhance the effect of chemical agent	Limited by wide applicability. Applicable to moving surfaces.

### Use of biological molecules

Use of biological molecules for antifouling applications are attractive as these are naturally occurring biomolecules or entities that are inherently less toxic, more efficient, and have greater specificity than many synthetic compounds [9]. Three of the most promising molecules are nitric-oxide releasing materials, peptides and peptoids.

#### Nitric oxide-releasing materials

**Fig. 1:**
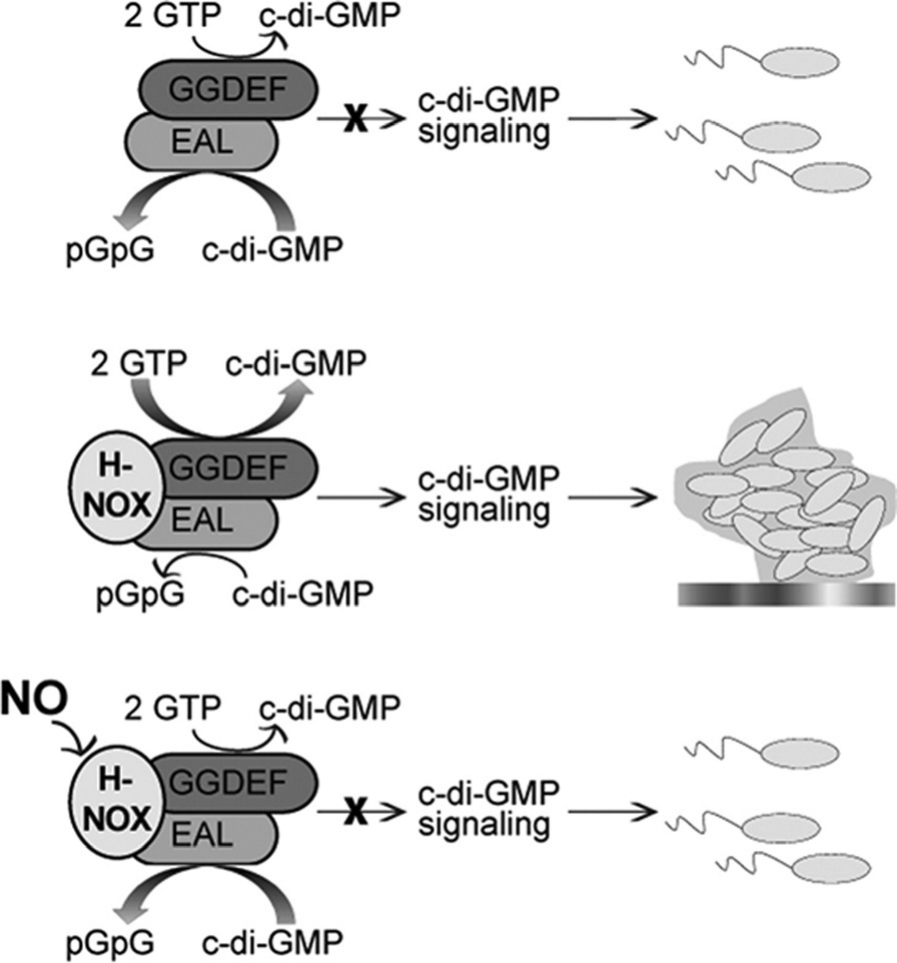
A model for NO regulation of c-di-GMP synthesis in *S. woodyi* suggested by Liu et al. Reproduced with permission from ref. [18] © American Chemical Society

Nitric oxide (NO) is a bactericidal agent. Unlike other germicidal release coatings such as silver nanoparticles and antibiotics, NO is naturally produced. It is an endothelium-derived relaxing factor that is responsible for regulating the natural homeostasis [13]. NO can disperse biofilm through a number of mechanisms including the production of oxidative or nitrosative stress-inducing moieties within the biofilm structure, bacteriophage induction and cell lysis [14]. For this reason, endothelium is one of the most thromboresisitive materials. Consequently, materials that can release NO at a steady-state equivalent to that released by the natural endothelium (0.5 × 10^−10^ mol cm^−2^ min^−1^) [15], can be a suitable material for making biomedical devices with enhanced antifouling capabilities [16, 17]. Liu et al. recently demonstrated that the presence of NO significantly reduces the formation of *Shewanella woodyi* (*S. woodyi*) biofilm by simultaneously down-regulating the cyclase activity and up-regulating the phosphodiesterase activity of the diguanylate cyclase gene (*Sw*DGC) (Fig. 1) [18]. In vivo *Sw*DGC and *Sw*H-NOX form a complex. In the absence of NO, *Sw*H-NOX is associated with *Sw*DGC and maintains its basal phosphodiesterase activity while enhancing diguanylate cyclase activity. Upon detection of NO, *Sw*H-NOX downregulates diguanylate cyclase activity and activates phosphodiesterase activity. Therefore, NO reduces the c-di-GMP concentration in *S. woodyi*, leading to a reduction in the extent of biofilm formation. This hypothesis suggests that NO can reduce the c-di-GMP concentration in *S. woodyi*, leading to a reduction in the extent of biofilm formation.

Initial studies using the NO-donor sodium nitroprusside (SNP) have shown that use of sub lethal concentrations (25 to 500 nM) of the NO-donor resulted in a significant dispersal of bacterial biofilms (*Pseudomonas aeruginosa*) [14]. In another study, researchers demonstrated that NO released from the donor PROLI/NO (1-[2-(carboxylato)-pyrrolidin-1-yl]diazen-1-ium-1,2-diolate) resulted in nearly 66 % reduction in proteins and nearly 50 % reduction in the biofilm surface coverage compared to the untreated control [19]. Similarly, using another NO-donor, *S*-nitroso-*N*-acetylpenicilamine (SNAP), Brisbois et al. successfully demonstrated a 90 % reduction in bacterial adhesion and infection by NO-releasing materials in a 7 days animal model [20].

**Fig. 2:**
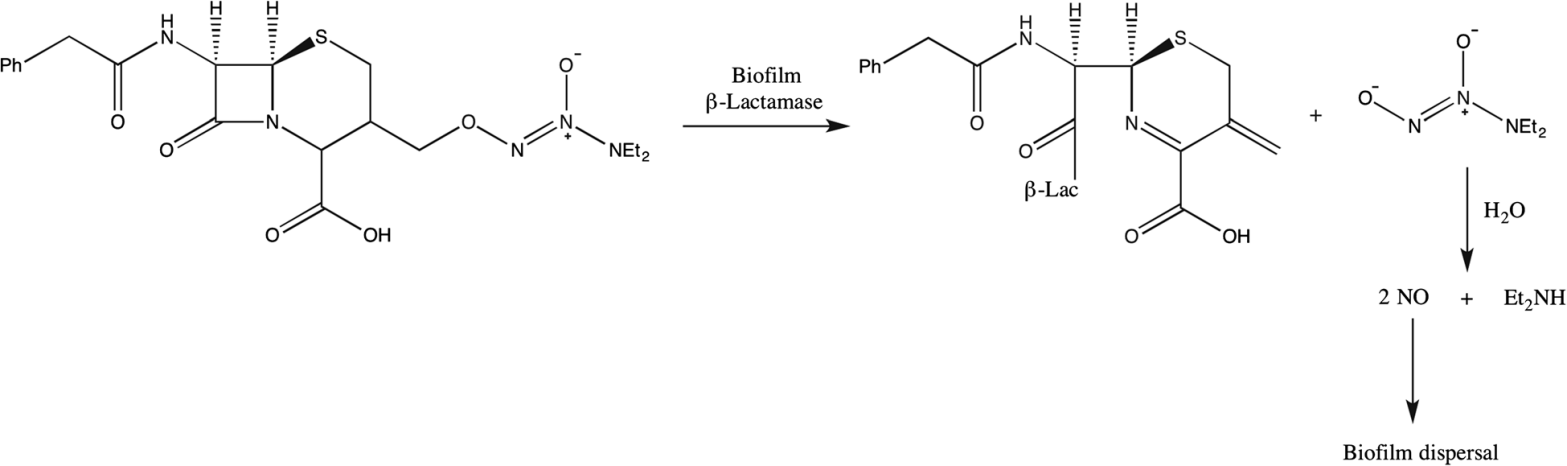
Mechanism of β-lactamase-triggered NO release and biofilm dispersion by cephalosporin-3′-diazeniumdiolate. Adapted from ref. [21]

From a clinical perspective, NO-releasing products can also provide potential advantages in controlling biofilm-related infections by releasing biofilm-specific enzymes such as β–lactamase. In one such attempt, Barraud et al. used cephalosporin-3′-diazeniumdiolate as the NO-donor pro-drug [21]. The β–lactam analog, cephalosporin, functioned as a protecting barrier for the NO-donor and the site specific NO-release was achieved upon specific activation by the bacterial enzyme β–lactamase (Fig. 2). This innovative pro-drug was found to be very effective in dispersing biofilms of various pathogenic species suggesting this as a potential precursor for developing anti-biofilm therapeutics.

Although NO can be an effective molecule for the prevention of biofilm formation and biofilm-related infections, its use in many biomedical applications remains a challenge because of high reactivity and short half-life,  its requirement for proper storage and delivery. Covalent incorporation of a suitable NO-donor during the synthesis or impregnating it into a suitable matrix during the processing are some of the approaches reported to date for modulating the NO-release towards biomedical applications [22–25]. One other limitation with the NO approach is that in certain instances NO was found to have no influence, and even stimulate biofilm formation in certain types of bacterial colonies [26–28]. Consequently, NO may be used selectively for controlling the biofilm formation in certain types of bacteria. Since there is a large range of biofilm responses, detailed research is required to establish the generalized use of NO over a variety of non-fouling applications.

#### Peptide and peptoid modified surfaces

Many peptide-based materials are used as antifouling agents because of their exceptional resistance to proteins including bovine serum albumin, fibrinogen, fibronectin, lysozyme, and streptavidin [29, 30]. Naturally occurring biomolecule such as amino acids, peptides, and polysaccharides are also commonly used in the development of innovative antifouling materials. Many of these biomolecules are believed to undergo structural reformations under physiological conditions to prevent biofouling [31]. Peptoids are non-natural biomimetic polymers. Unlike poly(ethylene glycol) (PEG) and zwitterions that will be discussed shortly, the surface structure and antifouling ability of the peptide- and peptoid-based materials can be tuned [31, 32]. Many known antifouling functionalities can be incorporated using a single backbone chemistry, and done so at precisely known locations, because of the sequence specificity of polypeptoids. A library of such sequence-specific materials can be designed for optimum performance.

In one example, a biomimetic antifouling material was prepared by Perrino et al. [33] by grafting dextran side chain to a poly(L-lysine) (PLL) backbone. The PLL-*graft*-dextran copolymer was found to have excellent antifouling properties to prevent nonspecific adsorption of proteins in a variety of architectures similar to a PEG-grafted analog. A similar biomolecule-based approach is to incorporate proteases onto the material surfaces. In one such attempt, Kim et al. [34] prepared protein resistant films by immobilizing hydrolytic enzymes pronase and α-chymotrypsin by sol–gel entrapment followed by covalent attachment to a polydimethylsiloxane (PDMS) matrix.

**Fig. 3:**
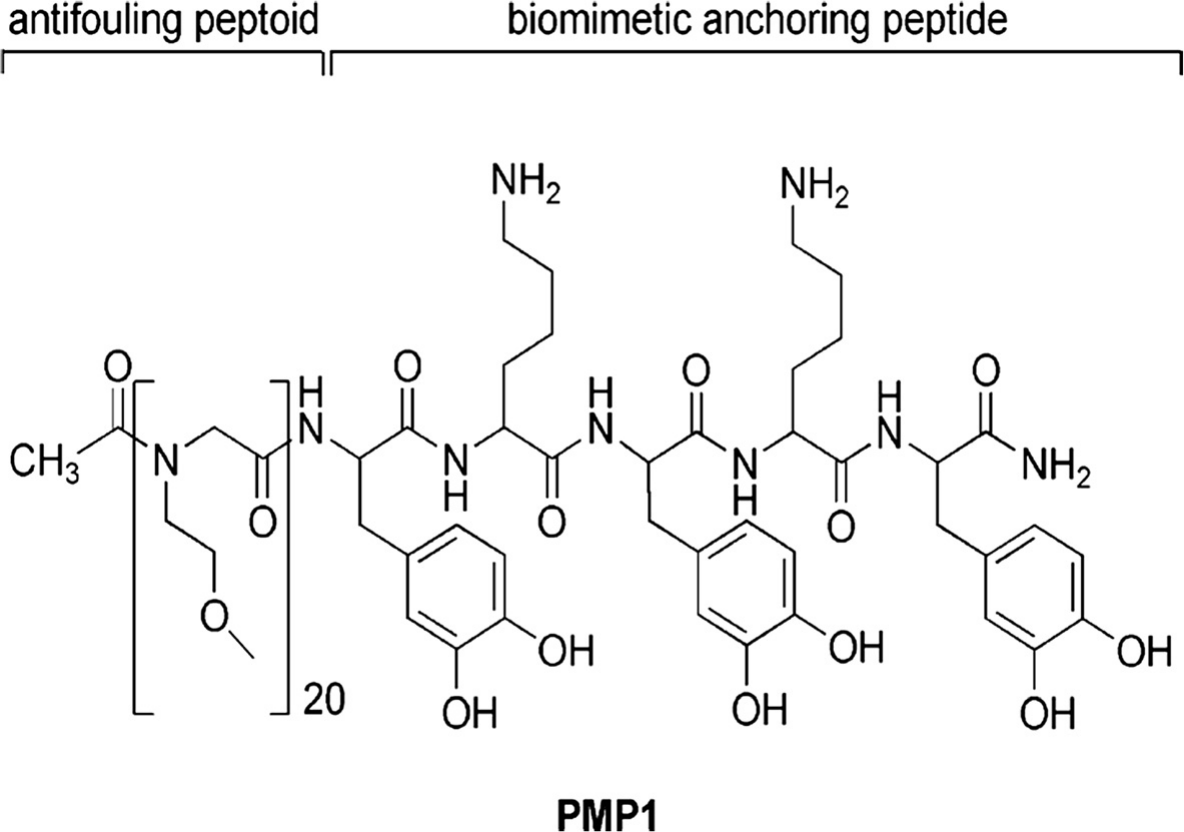
Structural details of a peptidomimetic polymer (PMP1). Reproduced with permission from ref. [36] © American Chemical Society

In another example, peptidomimetic synthetic approaches have been used to produce novel sequence-defined synthetic polymers that mimic the overall topology and biological activities of various natural peptides [35]. Using this method, polypeptoids (*N*-substituted glycines) have been synthesized for use as surface coatings with robust and long-term antifouling properties in biological environments. Figure 3 illustrates one example of this approach in which Statz et al. prepared a chimeric peptidomimetic peptide-peptoid based material (PMP1) using an *N*-substituted glycine and a short functional peptide [36]. The short functional peptide domain provided robust adsorption to various surfaces, while the peptoid oligomer provides resistance to protein and cell fouling. The material exhibited a significant reduction of serum protein adsorption in vitro and showed exceptional resistance to mammalian cell attachment for over five months. Poly(*β*-peptoids) (poly(*N*-alkyl-*β*-alanine)s) [37] also exhibited excellent resistant to nonspecific protein adsorption due to the strong hydrogen-accepting ability of these polymers under physiological conditions.

### Chemical modification of surfaces

Unlike the approaches discussed in a previous section, need exists for non-specific protein repulsion. This can be achieved by incorporating hydrophilic polymers such as PEG, amphiphilic fluoropolymers, zwitterionic polymers [6]. Chen et al. have summarized these polymers under two major classes, polyhydrophilic and poly zwitterionic [38]. While hydrophilic polymers resist protein adsorption by forming a hydration layer, hydrophobic surfaces prevent fouling by being able to readily release the adsorbed proteins and cells. All these polymers can be applied onto a variety of surfaces by one of many physical techniques such as spin-coating and dip-coating, or chemical methods such as covalent grafting. We will discuss the application of some of these polymers to biomedical devices.

#### Hydrophilic polymers

Surface modification with PEG is widely used in preventing biofouling on medical devices [39]. Each ethylene glycol repeating unit in the PEG backbone can strongly bind to one water molecule, bridging the ether oxygen along the 7_2_ helical PEG chain [40–42]. This unique interaction between the water and the PEG chain results in the formation of a highly hydrated layer and ultimately leads to a steric hindrance to the approaching protein molecules [38]. When a protein molecule approaches a hydration layer barrier, the resulting compression of the layer can decrease the conformational entropy of the polymer chains that ultimately lead to the repulsion of the approaching proteins. Such a hydration layer does not occur in polyoxymethylene even with its higher O/C ratio. High mobility and large exclusion volume of the PEG chains also contribute towards the overall antifouling characteristics of the polymer.

Despite the attractiveness of PEG as an antifouling agent, low surface densities [43, 44] and susceptibility to oxidative damages [45] limit their antifouling capabilities overlong-term applications. Additionally, it has been shown that reactive oxygen species, produced by PEG might modulate the cell response [46]. For these reasons, alternate hydrophilic polymers such as polyglycerols [47], polyoxazolines [48], polyamides [49], and naturally occurring polysaccharides [17] have been evaluated for antifouling applications.

#### Immobilization of PEG

A surface can be functionalized with PEG by using either adsorption of presynthesized PEG onto the surface (graft-to strategy), or by growing the polymer in-situ via surface adsorbed initiation group (graft-from strategy) [50]. Both of these techniques immobilize PEG on surfaces to confer them with protein and cell resistance. However, anchoring PEG by using functional groups on the surface requires extensive chemical modification of the surface, especially to make them less susceptible to thermal and hydrolytic degradation, and is therefore limited by the surface chemistry.

Following nature’s cues, PEG can be robustly anchored onto a variety of surfaces using a mussel-mimicking linker, adhesive L-3,4-dihydroxyphenylalanine (DOPA), a catechol functionality [51]. DOPA, DOPA peptides, or a catechol-mimic molecule can be covalently attached to the PEG terminal hydroxyl groups or to side chains. Alternatively, DOPA or a catechol-based monomer can be directly incorporated into the polymer backbone during polymerization. The catechol segment imparts the cohesive and adhesive characteristics, while PEG contributes to the antifouling characteristics [52].

**Fig. 4:**
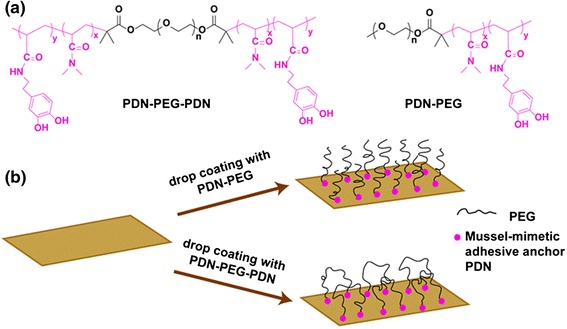
**a** Chemical structure of the triblock copolymer PDN-PEG-PDN and the diblock copolymer PDN-PEG. **b** Schematics of the preparation of surfaces bearing polymer brushes and polymer loops using drop coating method. Reproduced with permission from ref. [53] © Royal Society of Chemistry

PEG surface immobilization can be done through the formation of either polymer loops or brushes. In both these instances, PEGs with end-tethered or pendent functionalized with DOPA derivatives were used to target surface immobilization through stable anchoring. In a representative approach, Li et al. reported the successful evaluation of a catechol-functionalized PEG-based ABA triblock polymer loops for antifouling applications. It was shown that the polymer loops prepared from an ABA triblock copolymer, poly[(*N*,*N*-dimethylacrylamide)_15_-*co*-(*N*-3,4-dihydroxyphenethyl acrylamide)_2_]-*b*-poly(ethylene glycol)_90_-*b*-poly[(*N*,*N*-dimethylacrylamide)_15_-*co*-(*N*-3,4-dihydroxyphenethyl acrylamide)_2_] (PDN-PEG-PDN) had better antifouling properties than the polymer brushes prepared from its diblock analog, poly[(*N*,*N*-dimethylacrylamide)_15_-*co*-(*N*-3,4-dihydroxyphenethyl acrylamide)_2_]-*b*-poly(ethylene glycol)_45_ (PDN-PEG) (Fig. 4) [53]. The polymer loops showed a better adaptation towards the external compression exerted by the approaching proteins and reduced the protein penetration than the polymer brushes prepared from the diblock polymers with similar graft density. Therefore, such polymer loops with catechol-functionalities provide strong anchoring to surfaces. Because of the extremely low friction coefficient and enhanced inhibition of cell adhesion and proliferation, they provide a superior option for making ocular lenses and articular implants [54].

#### Zwitterionic polymers

Zwitterionic materials are amphoteric materials with both positive and negative charges. Because of the strong dipoles of the zwitterions and electrostatic itneractions, they have a stongly bound hydration layer that leads to “superhydrophilicity” and high protein resistivity [55]. Consequently, the extent of nonfouling characteristics exhibited by this group of materials is greater than that observed with hydrophilic/hydrophobic materials. Zwitterionic phospholipids such as phosphorylcholine constitute the outer surface of the nonthrombogenic erythrocyte cell membranes [56, 57]. Inspired by the antifouling properties of these naturally occurring membrane molecules, researchers have explored the potential utility of zwitterionic polymeric materials such as polybetaines or its structural analogs in a variety of biomedical applications, including nonthrombogenic surfaces [58].

An early example of bio-membrane mimicry is the preparation of phosphobetaine (PB)-based antifouling surfaces by incorporating the structural analogs of the naturally occurring lipid dipalmitoyl phosphatidylcholine (DPPC), such as diacetylenic phosphatidylcholine (DAPC) and methacryloyloxyalkyl phosphorylcholines (MAPC) [59–62]. Because of the characteristic ability of the phosphobetaines to retain a large amount of water with the zwitterionic head groups while retaining protein repulsion, PB-modified hydrogels are finding their importance in contact lens applications for improving the wettability and surface properties [63].

Other polybetaines, such as carboxybetaines (CB) and sulfobetaines (SB) are also been extensively evaluated for various nonfouling biomedical applications. Among these, CB is attractive because of its unique capability for immobilizing ligands such as proteins and antibodies, onto the carboxyl groups [64, 65]. Cheng et al. used a cationic precursor of poly(carboxybetaine methacrylate) (polyCBMA) to produce a switchable polymer surface coating with self-sterilizing and nonfouling capabilities [66, 67]. The cationic precursor of polyCBMA killed more than 99.9 % of *Escherichia coli* K 12 in 1 h and upon hydrolysis switched to a zwitterionic nonfouling surface with the release of more than 98 % of the dead bacterial cells and prevented any further protein attachment and biofilm formation.

Like many carboxybetaines, densely packed poly(sulfobetaine methacrylate) (polySBMA)-grafted surfaces have also been found to be completely resistant to the adsorption of a number of plasma proteins including human serum albumin, gamma globulin, fibrinogen, and lysozyme, even at low ionic concentrations [68–70]. PolyCBMA is also an interesting polymer for making blood-compatible surfaces, because of its structural comparability with the naturally occurring glycine betaine. Furthermore, the surface carboxyl groups polyCBMA provide a suitable platform for covalent immobilization of bioactive species such as monoclonal mouse antibody (mAb, anti-hCG) for specifically binding to human chorionic gonadotropin (hCG) while retaining the nonspecific protein repellency [71]. This dual functional behavior of polyCBMA makes it useful for the design of antifouling surfaces for biosensors and diagnostic applications.

#### Hydrophobic polymers

Hydrophobic coatings have been developed in textile industry to prevent staining [72]. These coating are effective as fouling release agents. In one study, Privett et al. reported the development of a superhydrophobic xerogel coating synthesized from a mixture of nanostructured fluorinated silica colloids, fluoroalkoxysilane, and a backbone silane [73]. The researchers showed a significant reduction in the adhesion of *Staphylococcus aureus* and *Pseudomonas aeruginosa* (*P. aeruginosa.)* on this fluorinated surface compared to their control samples. In another example, Li et al. [74] used a hydrophobic liquid-infused porous poly(butyl methacrylate-co-ethylene dimethacrylate) surface (slippery BMA-EDMA) for reducing the adhesion of *P. aeruginosa.* Only ~1.8 % of the slippery surface was covered by the environmental *P. aeruginosa* PA49 strain whereas the uncoated glass controls exhibited coverage of ~55 % under the same conditions. However, mainly due to the toxicity concerns, the hydrophobic polymers are not studied as extensively as their hydrophilic analogs.

Recently, Xue et al. reported the development of superhydrophobic antifouling poly(ethylene terephthalate) (PET) fabrics by chemical etching followed by grafting of fluorinated methacrylate polymers via surface-initiated atom radical polymerization (SI-ATRP) [72]. The hydrophobicity of the surfaces were controlled by tuning the polymerization time and a contact angle of nearly 160 ° was reported by the researchers after 8 h with excellent antifouling properties. Since PET is extensively used in a variety of biomedical applications, this innovative approach can be adapted to improve the antifouling properties of many currently available biomedical devices.

### Micropatterning of surfaces

#### Influence of surface texture

It has been known for more than 100 years that cell response is influenced by surface topology [75]. Investigations over the past 50 years have demonstrated that surface topography in the μm length scale affects the cell adhesion [76–81]. Many of these investigations were carried out in the context to cell survival and promotion of cell growth. Recent work has shown that such surface topography can be used reduce biofilm formation [82]. The discovery that such topography is in fact used by marine species to prevent biofouling has inspired the development of strategies for developing similar surfaces on biomedical devices [10, 83]. Topographical features suitable for controlling cell adhesion are traditionally produced by photolithography [82]. Alternative approaches include demixing [84–86], dewetting [87], solvent evaporation [84], and laser ablation [88]. These and other techniques can produce micro-topographies found on leaves on many plants, most well known being lotus, and dermal denticles on skins of marine organisms such as shark, whales, bottlenose dolphin mussels, snail shells, and edible crab.

The mechanisms by which the naturally occurring micro-topographic features prevent biofilm formation are still not clear. There are at least four mechanisms that contribute to the antifouling properties of the surface. In one mechanism, the lotus effect, superhydrophobity of the surface causes water to bead up on the surface, and pick up the contaminants as it rolls off the surface, and thus prevent attachment of any cells onto the surface [11, 89]. In the second mechanism, the topography is such that it prevents cellular attachment [90, 91]. In the third, the drag and the efficient flow of water, sloughs off the organism of the surface [10]. According to the fourth mechanism, marine animals secrete substances contribute to the antifouling properties of these surfaces [83, 92]. Irrespective of the mechanisms, these bioinspired structures, since they do not alter the chemical composition or add extraneous chemical into the mix, have found potential use in applications such as catheters [93]. We will discuss two bioinspired micropatterns that highlight this class of structures.

#### Superhydrophobic self-cleaning surfaces

**Fig. 5:**
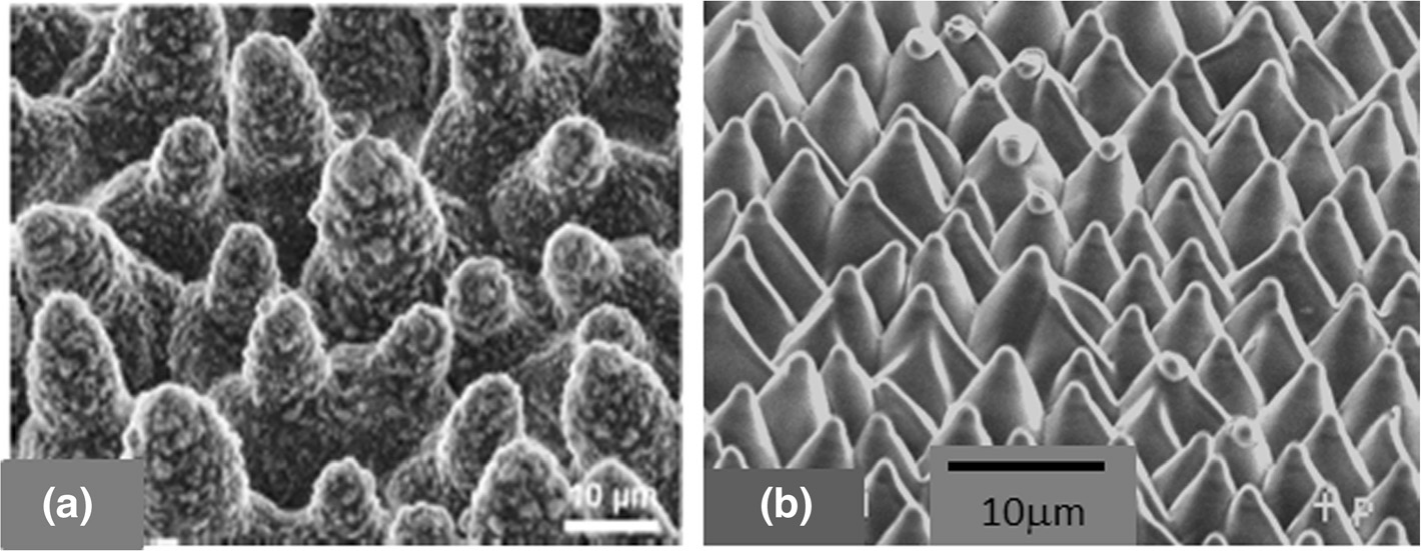
**a** The uniform conical cells on superhydrophobic leaves of a lotus plant. Reproduced from ref. [96] © Ensikat et al. **b** Uniform cones on a laser ablated film of poly(ethylene terephthalate) that are hydrophilic [88]

Water drops that fall upon the surface of leaves of certain plants such as that of lotus move freely on the surface, collect contaminants as they roll of the surface [94]. Such self-cleaning capabilities that results from the super-hydrophobicity has inspired the development of a similar architecture that can be imprinted on biomedical devices. This lotus effect arises from two levels of structure seen under electron microscope [93, 95, 96]. As seen in Fig. 5a, there are micro-scale mound like structures that are decorated with nano-scale hair-like structures. The air trapped in the cavities between the convex shaped cones minimize wetting making the surface hydrophobic. The hair like structure are hydrophobic hydrocarbon tubules. A combination of the micro-cones and waxy nano-structures make the surface superhydrophobic with contact angle between 90° and 150°. More importantly, the contact angles have very small hysteresis that allows the droplets to roll off the leaf and thus making the structure self-cleaning. Such surfaces can be produced by lithographic techniques [12, 89] as well as by self-assembly techniques [88].

It should be noted, that the 2^nd^ level of wax-like hairy is important for the superhydrophobicity of the surface. There are cone-like structures that lack the hair features present on the cones of lotus leaf. As a consequence, a droplet of water spreads on these leaves. Such structures are present on the leaves of plants such as Calathea Zebra [95]. The structure shown in Fig. 5b mimics these hair-less cones, and was obtained laser ablation of a polyester film [88]. These structures are formed by self-assembly, and can thus be formed on any complex surface found on medical devices.

#### Topography driven antifouling

**Fig. 6:**
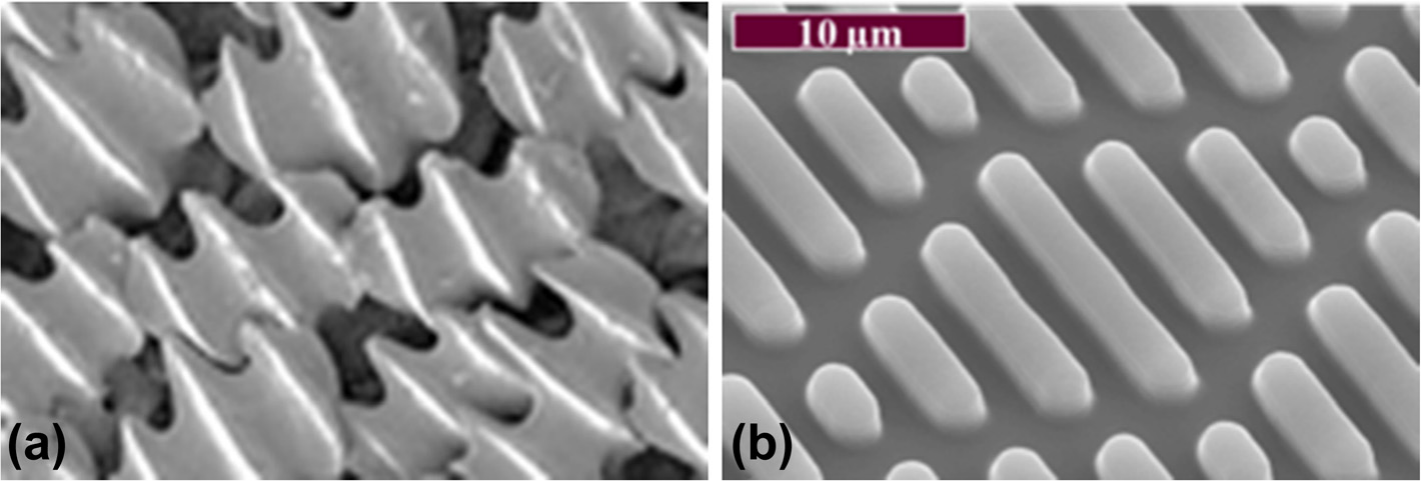
Sharklet technologies mimicking the micropatterns on Shark skin. **a** Skin of a Bull Shark © AMNH/R. Rudolph. Reproduced with the permission from the American Museum of Natural History (enlarged from the original). **b** Topography mimicked in the Sharklet™ surface technology. Reproduced with the permission of Prof. Brennan, Biomedical Engineering, University of Florida

One of the widely successful surface patterns for antifouling are inspired by shark skin is a combination of self-cleaning and low adhesion/drag surfaces (Fig. 6a). In general, the shape of the groves contribute to the low-drag and the self-cleaning properties of the shark skin. Analysis of the microtopographies present various marine species have suggested several key surface parameters that influence antifouling: low fractal dimension, high skewness of roughness and waviness, higher values of anisotropy, lower values mean roughness leading to improved antifouling characteristics [10, 97, 98]. Carman et al. [99] fabricated engineered multifeature topography in a polydimethylsiloxane elastomer to replicate the skin of fast moving sharks (Sharklet AF™) (Fig. 6b). Nearly 85 % reduction in the settlement of *Ulva linza* zoospores were achieved with the complex Sharklet AF™ topographies which consist of 2 μm wide engineered channels.

Although Micropatterned surfaces by themselves have been shown to reduce the transfer of bacterial contamination [100], there are other reports that suggest additional chemical modification may be necessary to achieve the desired level of antifouling [83]. For instance, mucous found on the shark skin also provides lubricating and antifouling benefits [92]. In another study, decoupling the surface chemistry from the topography lead to fouling of such surface during 3 to 6 weeks of immersion [101]. Such studies show that topographical features and surface properties can play a major role in designing antifouling surfaces for innovative biomedical surfaces [100].

The surface patterning involves surface modification via nanoparticles, photolithography, mesoporous polymers, or surface etching, sometimes in conjunction with additional chemical modifications to reduce surface energy. These often require harsh synthetic conditions complex fabrication techniques [89, 102, 103] thus limiting the substrate type and geometry that may be coated.

## Conclusions

Antifouling can be achieved by mimicking the strategies developed by nature instead of using synthetic antimicrobials and antibiotics. We have presented several strategies that can be adopted on biomedical devices and a comparative summary of various methods is presented in Table 1. Nitric oxide (NO) releasing agents are based on processed used by endothelium. Peptides and peptoids use specific protein repulsion approaches. There are widely successful approaches based on hydration layer that uses hydrophilic polymers such PEG and zwitterions. Several micropatterning approaches that mimic the surfaces of plant leaves and skins of marine animals are also discussed. These methods suggest the many ways in which the formation of biofilms on the material surface can be prevented in various biomedical devices particularly for blood-contacting biomedical applications.

## Abbreviations

BMA-EDMA, poly(butyl methacrylate-co-ethylene dimethacrylate); CB, carboxybetaine; c-di-GMP, cyclic diguanylate; DAPC, diacetylenic phosphatidylcholine; DOPA, 3,4-dihydroxyphenylalanine; DPPC, dipalmitoyl phosphatidylcholine; hCG, human chorionic gonadotropin; mAb, monoclonal antibody; MAPC, methacryloyloxyalkyl phosphorylcholine; NO, Nitric oxide; *P. aeruginosa*, Pseudomonas aeruginosa; PB, phosphobetaine; PDMS, polydimethylsiloxane; PDN-PEG, poly[(*N*,*N*-dimethylacrylamide)_15_-*co*-(*N*-3,4-dihydroxyphenethyl acrylamide)_2_]-*b*-poly(ethylene glycol)_45_; PEG, poly(ethylene glycol); PET, poly(ethylene terephthalate); PLL, poly(L-lysine); PMP1, a peptidomimetic peptide-peptoid based material; polyCBMA, poly(carboxybetaine methacrylate); polySBMA, poly(sulfobetaine methacrylate); PROLI/NO, 1-[2-(carboxylato)-pyrrolidin-1-yl]diazen-1-ium-1,2-diolate; *S. woodyi*, Shewanella woodyi; SB, sulfobetaine; SI-ATRP, surface-initiated atom radical polymerization; SNAP, *S*-nitroso-*N*-acetylpenicilamine; SNP, sodium nitroprusside; *Sw*DGC, diguanylate cyclase gene of *S. woodyi*; *Sw*H-NOX, *S. woodyi* with heme-nitric oxide/oxygen binding
